# Co-delivery of plantamajoside and sorafenib by a multi-functional nanoparticle to combat the drug resistance of hepatocellular carcinoma through reprograming the tumor hypoxic microenvironment

**DOI:** 10.1080/10717544.2019.1654040

**Published:** 2019-11-18

**Authors:** Ying Zan, Zhijun Dai, Liang Liang, Yujiao Deng, Lei Dong

**Affiliations:** Department of Digestive, The Second Hospital of Xi'an Jiaotong University, Xian, China

**Keywords:** Sorafenib, plantamajoside, drug resistance, nanoparticles, hypoxic microenvironment

## Abstract

Sorafenib (SOR) is a multi-kinase inhibitor that was approved as the first-line systematic treatment agent of hepatocellular carcinoma (HCC). However, the anti-cancerous effect of SOR is dramatically impaired by the drug resistance, insufficient accumulation at tumor tissues, and limited tumor inner penetration. To combat the above issues, the PLA-based nanoparticles were first fabricated and co-loaded with SOR and plantamajoside (PMS), natural herbal medicines that possess excellent anti-cancerous effect on many types of drug resistant cancers. Then, the polypeptide CT, which is tumor-homing and cell membrane penetrable, was further decorated on the dual-agents loaded nanoparticles (CTNP-PMS/SOR) to enhance tumor accumulation of drugs. Importantly, the CT peptide is a conjugate derived from the covalent conjugation of CVNHPAFAC peptide, a tumor-homing peptide, on the fourth lysine of TAT, namely cell membrane penetrating peptide, through a pH-sensitive hydrazone bond. By this way, the cell penetrating ability of TAT was dramatically sealed under the normal condition and immediately recovered once the nanoparticles reached tumor sites. Both *in vivo* and *in vitro* experiments demonstrated that the anti-cancerous effect of SOR on malignant HCC was significantly enhanced after co-loaded with PMS. Mechanisms studies revealed that the PMS is capable of reprograming the tumor hypoxic microenvironment, which represents the main cause of drug-resistance of tumor cells. Besides, functionalization of the NP-PMS/SOR with CT peptides signally improved the accumulation of drugs at tumor sites and penetration of agents into tumor cells, which in turn resulted in stronger capacity of tumor growth inhibition.

## Introduction

Liver cancer has been the leading cause of cancer-related death, with an overall 5-year survival rate below 10% (Wang et al., 2016; Dong & Zhang, 2018). As the most dangerous and common liver cancer, hepatocellular carcinoma (HCC) is accounting for 90% of all primary hepatoma (Jeon et al., 2018). However, the present therapy options for clinic treatment of HCC are not sufficient and hardly satisfactory. Although a wide array of novel small molecule drugs have been developed, tumor drug resistance significantly impaired the therapy effect (Aghazadeh & Yazdanparast, 2017). The hypoxic microenvironment in the solid tumor tissues and/or within the tumor cells represents one of the primary causes of drug resistance (Gombodorj et al., 2017). Such unique microenvironment at tumor site is mainly formed by the complex formation mechanisms of tumor tissues, such as overgrowth of the cancer cells, blood abnormalities, and overlong oxygen diffusion distance (Gombodorj et al., 2017; Qin et al., 2018). Previous studies have demonstrated that the hypoxia inducible factor (HIF) is the key gene regulatory factor that was involved in cell hypoxia response, and over expressions of HIF-1 and HIF-2 is closely related to chemoresistance of tumor cells (Harashima et al., 2017; Nouri et al., 2019).

Besides the hypoxic microenvironment, insufficient tumor accumulation of chemotherapeutics represent another severe obstacle to achieve a relatively satisfactory treatment effect (El-Housiny et al., 2018; Wang et al., 2019). Additionally, majority of conventional chemotherapeutic agents such as doxorubicin results in nonspecificity and easily lead to serious cardiotoxicity (Ghibu et al., 2012). To overcome such dilemma, nanoparticles-based active targeting has emerged as a robust and promising strategy (Zhao et al., 2018). Active targeting of nanoparticles to cancer cells can be achieved using ligands that bind specifically to certain biomolecules that are overexpressed on the surface of cancer cells (Lin et al., 2018). Due to their specificity, these ligands act as a homing device and direct the nanoparticles to specific cancer cells. CVNHPAFAC-NH2 peptide is a tumor-homing molecule that selectively binds to human sonic hedgehog (Matsuo et al., 2011). Aberrant activation of the pathway of SHH/Gli1 was considered closely associated with various tumorigenesis including liver cancer, pancreas, melanoma, colorectal, and prostate carcinomas (Gan et al., 2016; Sun et al., 2017; Yang et al., 2018). Additionally, the sonic hedgehog was abundant in the tumor microenvironment and always involved in the tumor angiogenesis and overexpressed in hepatoma carcinoma cell (Chen et al., 2017).

Although modification of tumor-homing peptides significantly enhanced the accumulation of drugs at tumor sites, efficient penetration of chemotherapeutics is another important prerequisite to the achievement of successful targeted anticancer agents (Song et al., 2016). Based on this, conjugation of tumor targeting peptides (TTPs) and cell penetrating peptides (CPPs) has been developed since it perfectly makes a combination of tumor specificity of TTPs and penetrating capacity of CPPs (Myrberg et al., 2008). TAT peptide is the most efficient family of CPPs (Koren & Torchilin, 2012). Conjugation of TAT has been shown to enhance the cellular uptake of doxorubicin-loaded liposomes, as well as improving transfer across the blood–brain barrier in *in vitro* models (Weng et al., 2019). However, the TAT peptide lacks tumor cell-specificity, which can lead to serious toxicity to normal tissues (Weng et al., 2019). Besides, the TAT peptides can result in endocytosis, which in turn accelerates elimination through the mononuclear phagocyte system (Qin et al., 2011). Previous studies demonstrated that the transmembrane transport capacity of TAT can be dramatically decreased by sealing of the fourth lysine and immediately recovered once uncovering the functional group (Liu et al., 2014). Such approach might provide a promising strategy for preferably use of TAT.

Recently, combination therapy of natural bioactive agent and chemotherapeutics has attracted increasing attention in combating many types of cancers for unique advantages of certain natural agents, such as high anti-tumor efficacy, multi-target inhibition, and ability of regulating tumor microenvironment (Jiao et al., 2019). For example, the natural products, oridonin and curcumin, have been recently used to enhance the anti-tumor effect of doxorubicin and paclitaxel, respectively (Yao et al., 2017; Zhang et al., 2017; Li et al., 2019). In the present study, we select the sorafenib (SOR), a broad spectrum kinase inhibitor that was approved for treating patients with unresectable HCC (Jindal et al., 2019), as the model drug. As the ATP-competitive kinase inhibitor, SOR is demonstrated to be able of targeting multiple ligands, including the BRAF, CRAF, MAP, kinases, VEGFR, and PDGFR (Wang et al., 2018). By the specific binding, SOR results in tumor cell apoptosis and disruption or inhibition of angiogenesis (Wang et al., 2018). However, previous study uncovered that overexpression of HIF-1α significantly impaired the anti-cancerous effect of SOR by inducing drug resistance (Long et al., 2019). Plantamajoside (PMS) is an extract from Herba Plantaginis with the role of antiviral, diuretic, antioxidant, and immune enhancement (Li et al., 2018). Previous studies have demonstrated that PMS possesses excellent anti-cancerous effect on many types of drug resistant cancers by complex mechanisms (Pei et al., 2015). Therefore, to achieve the goal of reducing therapeutic resistance, the PLA nanoparticles was developed here and co-loaded with PMS and SOR (NP-PMS/SOR). For enhancing the tumor targeting efficacy and reducing unwanted accumulation at normal tissues, the surface of NP-PMS/SOR was decorated with a polypeptide CT (CTNP-PMS/SOR). The CT peptide was developed by conjugation of CVNHPAFAC on the fourth lysine of TAT by a pH-sensitive hydrazone bond. By this way, the developed CTNP-PMS is supposed to be safety enough under normal physiological conditions and can exert its excellent anti-cancerous effect in the acidic tumor microenvironment.

## Materials and methods

### Materials, cells, and animals

Methoxy-poly (ethylene glycol)-poly (lactic acid) (mPEG-PLA, 33,000 Da) and maleimide-poly (ethylene glycol)-poly (lactic acid) (Mal-PEG-PLA, 34,000 Da) were obtained from Adamas Corporation (Shanghai, China). The SOR and PMS were obtained from Melonepharma (Dalian, China) while the 3-(4,5-dimethyl-2-thiazolyl)-2,5-diphenyltetra-zoplium bromide (MTT) and fluorescein isothiocyanate (FITC) were purchased from Beyotime (Haimen, China). The CVNHPAFAC peptide, TAT (GRKKRRQRRRC) peptide, and the polypeptide CT were synthesized by China Peptides Co., Ltd. (Shanghai, China). The primary anti-bodies and the fluorescent-labeled correspondence were obtained from Santa Cruz (Shanghai, China). The horseradish peroxidase (HRP)-conjugated anti-rabbit or anti-mouse secondary antibodies were purchased from Thermo (Shanghai, China). Dulbecco’s modified Eagle medium (DMEM) medium, fetal bovine serum (FBS), and trypsin–EDTA solutions were purchased from Gibco (Carlsbad, CA).

The human liver cancer cell line (HepG2) was obtained from Chinese Academy of Sciences Cell Bank and cultured in DMEM containing 10% FBS supplemented with 100 U/mL penicillin and 100 µg/mL streptomycin. The hypoxic condition of the HepG2 cells was obtained by incubating the cells in a CO_2_ incubator with 94% N_2_, 5% CO_2_, and 1% O_2_ (Qin et al., 2018). To ensure the cancer cells were chemotherapeutic-resistant, the HepG2/SOR cells were incubated in complete 1640 medium containing 0.5 μM SOR for one week before they were subjected to experiments. By this way, the SOR-resistant HepG2 cells, named as HepG2/SOR cells, were established. Male nude Balb/c mice (18–20 g) were obtained from Shanghai Sino-British Sippr/BK Lab Animal Ltd. (Shanghai, China) and were maintained at a constant temperature (25 ± 1 °C). The animal studies were conducted in accordance with the protocols approved by Institutional Animal Care and Use Committee (IACUC), School of Pharmacy.

### Preparation and characterization of nanoparticles

The multi-functional nanoparticles CTNP-PMS/SOR was developed by the reported emulsion and solvent evaporation method (Feng et al., 2015). In brief, the blend of PMS (0.25 mg), SOR (0.25 mg), Mal-PEG-PLA (2.5 mg), and MPEG-PLA (22.5 mg) was dissolved in 2 mL dichloromethane (DCM) and then added with 4 mL of 1% (w/v) sodium cholate solution. To form the oil-in-water (o/w) emulsion, the mixtures above were subjected to sonication at 240 W for 80 s. The obtained nanoparticles solutions were subsequently poured into 50 mL of 0.5% sodium cholate solution under gently stirring by a magnetic stirrer. After an incubation of 10 min, the solutions were evaporated by a rotary vacuum to completely remove the residual organic solvent. Finally, the drug-loaded nanoparticle (NP-PMS/SOR) was collected by centrifugation under the condition of 14,500 rpm at 4 °C for 45 min. For peptides functionalization, the achieved NP-PMS was resuspended with 2 mL of distilled water and poured into a penicillin bottle. Then, different peptide solutions including CVNHPAFAC, TAT, and CT were respectively added into the penicillin bottle at 1.5:1 molar ratio of peptide to maleimide. After gently stirring for 6 h, the peptides functionalized nanoparticles (CVNHPAFACNP-PMS/SOR, TATNP-PMS/SOR, and CTNP-PMS/SOR) were concentrated by centrifuging for 45 min at the speed of 14,000 rpm.

The particle sizes and zeta potential of the nanoparticles were determined using the Malvern Zetasizer (Malvern, nanoZS, Worcestershire, UK), and the morphological examination was performed using transmission electron microscopy (TEOL2010, JEM). The encapsulation efficiency of the LHNPs was measured using high performance liquid chromatography (HPLC) method.

### Cell uptake assay

5 × 10^3^ HepG2/SOR cells were cultured in each well of the 96-well plates and allowed to grow for overnight. Then, various FITC-labeled nanoparticle formulations were respectively added into the plates at the concentration of 200 µg/mL. After co-incubation for 1 h, the nanoparticle solutions in the plates were removed and cells were washed three times with PBS. Then, the cells were trypsinized and collected by centrifugation before being determined by a flow cytometer (FCM). For qualitative analysis, 4% paraformaldehyde was added into each well of the plates after the cells were washed by PBS. Then, the fluorescent intensity of cells was observed under a fluorescence microscope.

### Cell proliferation analysis

Cell proliferation was analyzed using the MTT assay according to the manufacturer’s instructions. 5 × 10^3^ HepG2/SOR cells in 100 µL DMEM were cultured in 96-well plates for overnight. Then, various nanoparticle formulations, including free SOR, NP-SOR, NP-PMS, NP-PMS/SOR, CVNHPAFACNP-PMS/SOR, TATNP-PMS/SOR, and CTNP-PMS/SOR, were added into each well. The concentrations of SOR were ranging from 1 to 1 × 10^3^ ng/mL. After incubation for 48 h, each well of the plates was supplemented with 10 μL of MTT (5 mg/mL) and allowed to react for 4 h. Subsequently, the spectrometric absorbance at 570 nm was measured with the microplate photometer (BioTek, Synergy2, Winooski, VT) after 150 μL of dimethyl sulfoxide (DMSO) was added to dissolve the formazan crystals.

### Wound-healing assay

For wound-healing assay, 1 × 10^4^ HepG2/SOR cells in 1 mL medium were plated in the each well of the six-well plates. After the cells were cultured to 90% confluence, a wound line was created in each well by a plastic pipette tip. Then, the produced cell debris was completely removed by washing with PBS for three times. Subsequently, 1 mL of fresh medium without serum and containing various formulations, including free SOR, NP-SOR, NP-PMS, NP-PMS/SOR, and CTNP-PMS/SOR, was supplemented and co-incubated with cells for 24 h. Thereafter, the cells that invaded into the wound surface were photographed by an inverted microscope (Olympus IX70, Tokyo, Japan) and the wound healing rate was calculated by the formula: *W*_E_/*W*_C_. The *W*_E_ represents the wound width of the experimental group while the *W*_C_ is the wound width of the control group.

### Migration assay

To perform the transwell migration assays, 5 × 10^4^ HepG2/SOR cells in 100 µL medium without serum and containing various formulations were seeded into the upper chamber of 24-well transwell with a non-coated membrane. The formulations including free SOR, NP-SOR, NP-PMS, NP-PMS/SOR, and CTNP-PMS/SOR, with the concentration of PMS or SOR was set at 200 ng/mL. In the meanwhile, the cells without any treatment were used as the control. Of great importance, the lower chamber of the 24-well transwell was covered with fresh medium with 10% FBS. After an incubation for 24 h, the cells that migrated from the upper chamber were fixed by 4% paraformaldehyde followed by staining with crystal violet. Then, the migrated cells were qualitatively analyzed under an inverted microscope and quantified using the microplate reader at 570 nm.

### Biodistribution studies

To evaluate the tumor-targeting efficacy of each nanoparticle formulation, the HepG2/SOR tumor-bearing mice were established first. In brief, 100 µL of HepG2/SOR cells suspension with the density of 1.0 × 10^5^/µL was injected into the right flank of BALB/c mice. Then, the tumor-bearing mice were kept under the standard condition with free access to food and water. When the tumors were grown to ∼100 mm^3^, 12 tumor-bearing mice were randomly divided into four groups (*n =* 3) and respectively injected with NP-PMS/SOR, CVNHPAFACNP-PMS/SOR, TATNP-PMS/SOR, and CTNP-PMS/SOR. Subsequently, the mice were sacrificed, and blood and tissues (heart, liver, spleen, lungs, kidneys, and tumors) were collected at 2, 6, 12, and 24 h after drug administration. SOR concentrations in plasma and tissue samples were analyzed by LC/MS/MS.

### *In vivo* antitumor effect

The randomly grouped (*n* = 9) tumor bearing-mice were intravenously injected with free SOR, NP-SOR, NP-PMS/SOR, CVNHPAFACNP-PMS/SOR, TATNP-PMS/SOR, and CTNP-PMS/SOR, respectively, with the PBS treated group as the control. Importantly, all of the mice were injected with SOR formulations every two days for a total of four injections with SOR dosage of 5 mg/kg. Then, the body weight of each mouse was carefully monitored every two days over a whole period of 14 days and the tumor volume was calculated using the formula: *V* = (*L*×*W*^2^)/2 (*L*: the longest dimension; *W*: the shortest dimension). All mice were sacrificed on day 14 after the first treatment and all of the tumor xenografts were excised and weighed. For further examination, tumors were fixed with 4% paraformaldehyde for 48 h and embedded in paraffin followed by being sectioned at 3-µm thickness. Finally, the slices were subjected to hematoxylin and eosin (H&E) analysis and TUNEL experiments for determination of the tumor cell necrosis and apoptosis. For the survival monitoring, the randomly grouped tumor-bearing mice (*n* = 6) were treated with various SOR formulations as above and the overall survival was carefully recorded.

### Western blot analysis

Total protein samples in cells or tumor tissues were extracted by the RIPA buffer (BOSTER, AR0105, Wuhan, China). Then, the protein samples were collected using the centrifugation method (14,000 rpm, 30 min) and the concentration of these protein samples was determined using the BCA method (Pierce, Rockford, IL). After that, each sample was separated on 10% SDS-PAGE gels followed by transferring to polyvinylidene difluoride (PVDF, Millipore, Billerica, MA) membrane. The samples were blocked with 5% nonfat dried milk for 1 h and subsequently incubated with primary antibodies against HIF-1α, PCNA, Caspase-3, and GAPDH at 4 °C overnight. After an overnight of incubation, all membranes were incubated with goat anti-rabbit IgG (1:1000, LK2003L, Sungene Biotech Co., Ltd, Tianjin, China) for 1 h at room temperature. Finally, the expressions of various proteins were visualized using ECL Western Blotting Substrate (Invitrogen, 32109, Carlsbad, CA).

### Statistical analysis

The data are reported as the mean ± standard deviation (SD). For comparison of the two study groups, statistical analysis was performed using Student’s *t*-test. Differences among three groups were analyzed using Fisher’s least significant difference (LSD) method. Values of *p* < .05 were regarded as statistically significant.

## Results

### PMS signally enhanced the cytotoxicity of SOR to drug resistant HepG2/SOR cells

**Figure 1.**
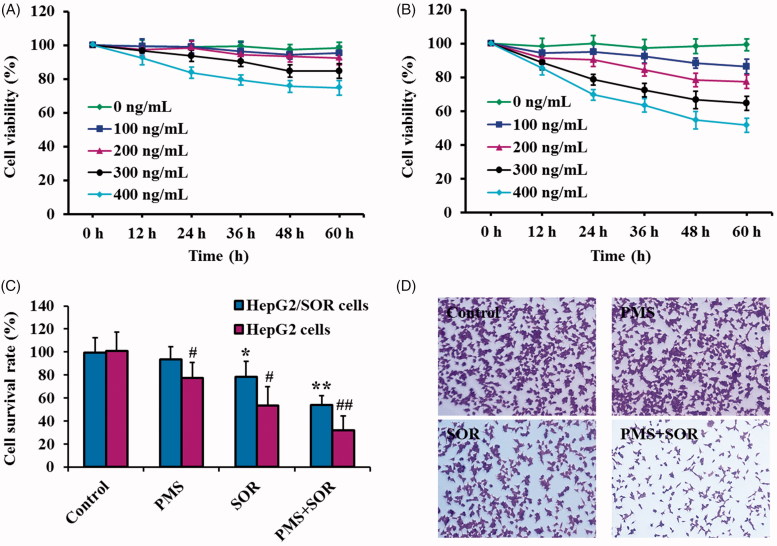
Evaluation of the anti-cancerous effect of PMS and SOR on HepG2/SOR cells *in vitro*. (A) Cell viability of HepG2/SOR cells post treating with different concentration of PMS and co-incubation for various times. (B) Cell viability of HepG2/SOR cells after incubation with different concentration of SOR and for various times. (C) Cytotoxicity of PMS and SOR to the sensitive HepG2 cells and SOR-resistant HepG2/SOR cells. (D) Cell cloning evaluation on HepG2/SOR cells post treatment with 200 ng/mL PMS, 200 ng/mL SOR, and PMS + SOR (both drugs were set at 200 ng/mL). *p < .05, **p < .01, compared with the control group of HepG2 cells. ^#^*p* < .05, ^##^*p* < .01, compared with the control group of HepG2/SOR cells.

To explore an appropriate treatment dose and incubation time of PMS and SOR, the HepG2/SOR cells were respectively exposed to different concentration of PMS and SOR. After 0, 12, 24, 36, 48, or 60 h treatment, the cell viability was determined by CCK8 kit. As shown in Figure 1(A), cells did not exhibit significant decrease in viability than that of control group under the drug concentration of 200 ng/mL or below, even co-incubation cells for 60 h. However, the cells incubated with 200 ng/mL of SOR exhibited significant lower cell viability when compared with the control cells and the viability is deceased with increasing the time of incubation (Figure 1(B)). Importantly, prolonging the incubation time to 60 h did not significantly enhance the cytotoxicity of PMS or SOR when compared with the cells incubation for 48 h. Giving these results together, the treatment dose of PMS and SOR for cell therapy was set at 200 ng/mL, while the incubation time was set at 48 h.

Subsequently, the effect of combinational therapy of PMS and SOR on HepG2/SOR cells was evaluated and compared with the effect on nonresistant cells. As shown in Figure 1(C), HepG2/SOR cells treated by SOR displayed a higher cell survival rate than that of the nonresistant cells, confirming that the established HepG2/SOR cells were exactly resistant to SOR. Of great importance, when compared with the cells only treated by SOR, the viability of HepG2/SOR cells was dramatically decreased post co-incubation with PMS + SOR, demonstrating that the PMS could signally enhance the cytotoxicity of SOR to drug resistant HepG2/SOR cells. Such results could be further confirmed by the cell colony assays, with the crystal violet staining results suggested that the colony formation of HepG2/SOR cells was inhibited more effectively by combination therapy of PMS and SOR than the monotherapy of SOR (Figure 1(D)).

### Characterization of nanoparticles

**Figure 2.**
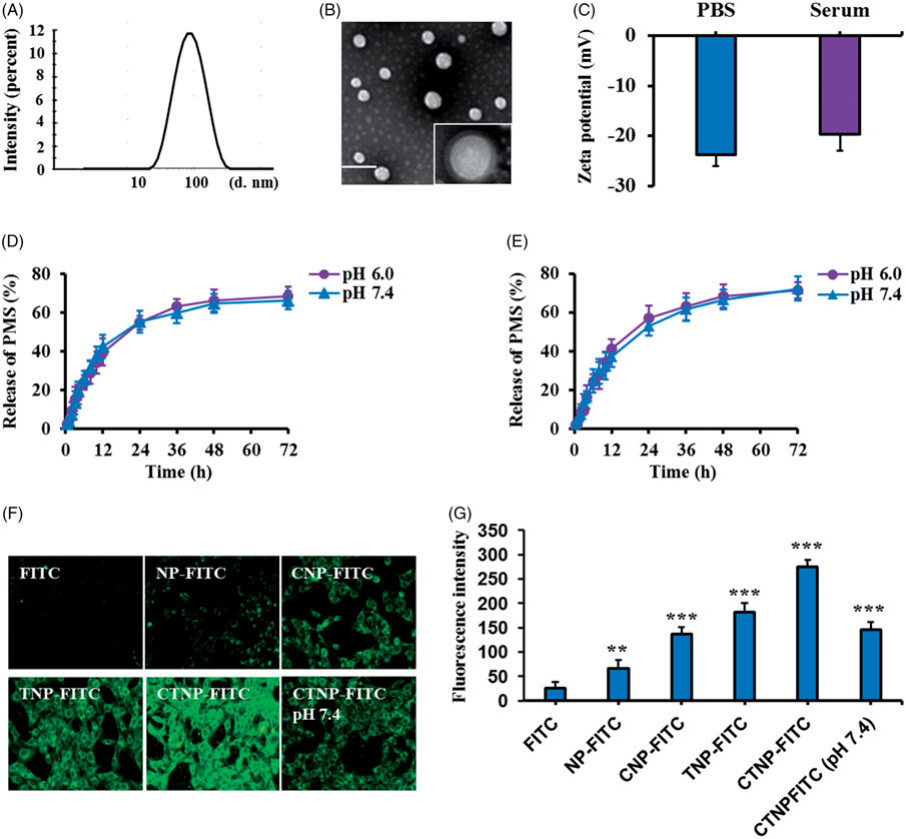
Characterization of CTNP-PMS/SOR and evaluation of cellular uptake of FITC-labeled various nanoparticle formulations. (A) Particle size distribution of CTNP-PMS/SOR determined by the DLS analysis. (B) TEM images of CTNP-PMS/SOR after negative staining with phosphotungstic acid. (C) Zeta-potential of CTNP-PMS/SOR after respectively incubated in PBS and serum-contained PBS for 24 h. Release behavior of PMS (D) and SOR (E) in the medium of pH 6.0, represents the tumor microenvironment, and pH 7.4, represents the normal physical condition. Qualitative analysis (F) and quantitative evaluation (G) of cellular uptake of different nanoparticles (labeled by the FITC) and compared with the free fluorochrome. ***p < .001, significantly higher than the cells treated by free FITC.

DLC analysis exhibited that the average size of CTNP-PMS/SOR was 109.23 nm (Figure 2(A)). TEM images of the developed CTNP-PMS/SOR showed the nanoparticles have an almost spherical morphology and core–shell structure (Figure 2(B)). The zeta potential was subsequently determined and results showed a value of –23.23 mV. Importantly, co-incubation of CTNP-PMS/SOR with rat serum for overnight did not affect the zeta potential, indicating the developed nanoparticles possess a good stability (Figure 2(C)). The loading efficiency (LE) and loading capacity (LC) of drugs have been further calculated, with results were 65.76% and 5.37% for PMS, and 68.31% and 6.13% for SOR. For comparison, the nanoparticles undecorated with CT peptides were also subjected to physicochemical properties evaluation as above. As results shown that, the particle size of NP-PMS/SOR was 77.86 nm, with the LE and LC were 66.32% and 5.64% for PMS while 67.34% and 7.12% for SOR. These results suggesting that the decoration of nanoparticles with CT peptides affect negligible on the physicochemical properties of NP-PMS/SOR.

The drug release behavior of PMS and SOR was studied in two kinds of medium, with the pH 6.0 representing the tumor microenvironment while the pH 7.4 represents the normal physical condition. Moreover, release behaviors of PMS and SOR were respectively investigated. Results shown in Figure 2(D,E) revealed that the CTNP-PMS/SOR exhibited similar and controlled release pattern in the two kinds of medium for both of PMS and SOR.

### Intracellular uptake of nanoparticles

To evaluate whether modification of CT peptide could enhance the accumulation of nanoparticles in tumor cells, HepG2/SOR cells were respectively treated with different nanoparticles which were labeled by FITC. As shown in Figure 2(F,G), a similar fluorescent intensity was observed in the cells treated by CNP-FITC and unmodified nanoparticles. It was mainly because the C peptide is not affinity to HepG2/SOR cells. However, the signal was dramatically elevated after cells treated by TNP-FITC, demonstrating a superior cell penetrating ability of TAT peptide. Of great importance, the cell penetrating ability of TAT was significantly down-regulated after sealing its functional group by conjugation with C peptides. As demonstrated, cells incubated with CTNP-FITC displayed a similar fluorescent intensity to the cells treated by TNP-FITC. Interestingly, under the condition of pH 7.4, the fluorescent signal of cells treated by CTNP-FITC did not show obvious difference from that of control or cells treated by CNP-FITC. It was ascribed to the fact that the linkage between C and TAT was acidic-sensitive and remain structural intact under the normal physiological condition while disrupt in the tumor acidic micro environment.

### Cytotoxicity of nanoparticles

**Figure 3.**
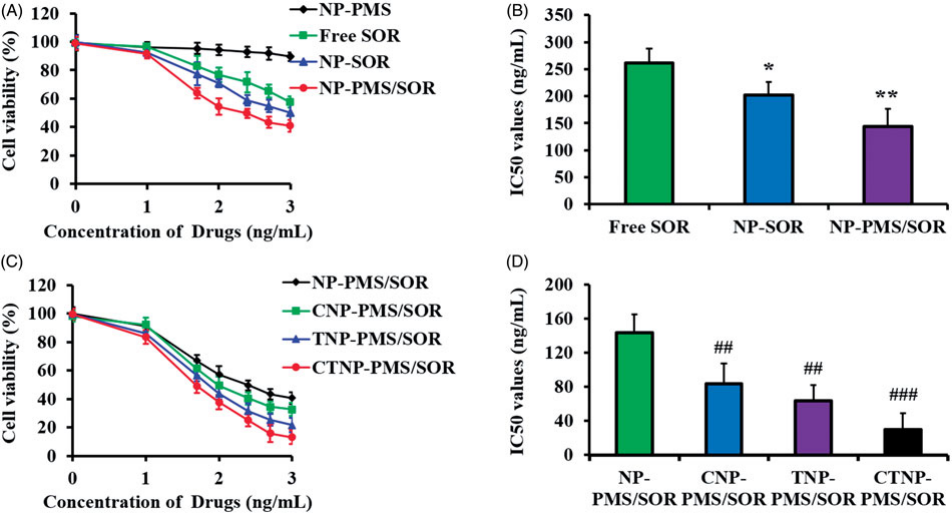
Cytotoxicity of different nanoparticle formulations to HepG2/SOR cells by MTT experiments. (A) Cell viability of HepG2/SOR cells after treated by NP-PMS, free SOR, NP-SOR, and NP-PMS/SOR for 48 h. Concentration of PMS or SOR was set at 200 ng/mL. (B) IC_50_ values of NP-PMS, free SOR, NP-SOR, and NP-PMS/SOR after the MTT experiments. (C) Cell viability of HepG2/SOR cells after treated by NP-PMS/SOR, CNP-PMS/SOR, TNP-PMS/SOR, and CTNP-PMS/SOR for 48 h. Concentration of PMS or SOR was set at 200 ng/mL. (D) IC_50_ values of NP-PMS/SOR, CNP-PMS/SOR, TNP-PMS/SOR, and CTNP-PMS/SOR after the MTT experiments. *p<.05, **p<.01, compared with the cells treated by free SOR. ^#^*p* < .05, ^##^*p* < .01, ^###^*p* < .001, compared with the cells treated by NP-PMS/SOR.

As demonstrated by the MTT assay results (Figure 3(A)), there was no significant cell viability decrease in the HepG2/SOR cells treated by NP-PMS when compared with the control group. However, the cytotoxicity of SOR was obviously enhanced after it was loaded into the PLA nanoparticles. Interestingly, the viability of the cells incubated with NP-PMS/SOR is markedly lower than that of the cells only treated by NP-SOR, confirming that the PMS is favorable to enhance the cytotoxicity of SOR. The IC_50_ value of each SOR formulation was as follows: 261.37 ng/mL for free SOR, 202.36 ng/mL for NP-SOR, and 143.57 ng/mL for NP-PMS/SOR.

To further improve the anti-cancerous effect of NP-PMS/SOR, various peptides were decorated on its surface. The cytotoxicity of these nanoparticle formulations was also determined using the MTT method. As shown in Figure 3(B), the anti-cancerous effect of NP-PMS/SOR was dramatically improved after modification of the tumor-homing peptide CVNHPAFAC. Of great importance, the TAT peptide functionalized NP-PMS/SOR leads to obviously lower cell viability than the CVNHPAFACNP-PMS/SOR, suggesting a superior cell membrane penetrating ability of TAT. Similar to the cellular uptake results, the HepG2/SOR cells exhibited the highest sensitivity to the polypeptide CT decorated nanoparticles (CTNP-PMS/SOR), with the IC_50_ value was 29.46 ng/mL, which was signally lower than that of the TATNP-PMS/SOR (63.74 ng/mL), CVNHPAFACNP-PMS/SOR (83.49 ng/mL), and NP-PMS/SOR (143.57 ng/mL).

### Inhibition of cell migration and invasion by CTNP-PMS/SOR

**Figure 4.**
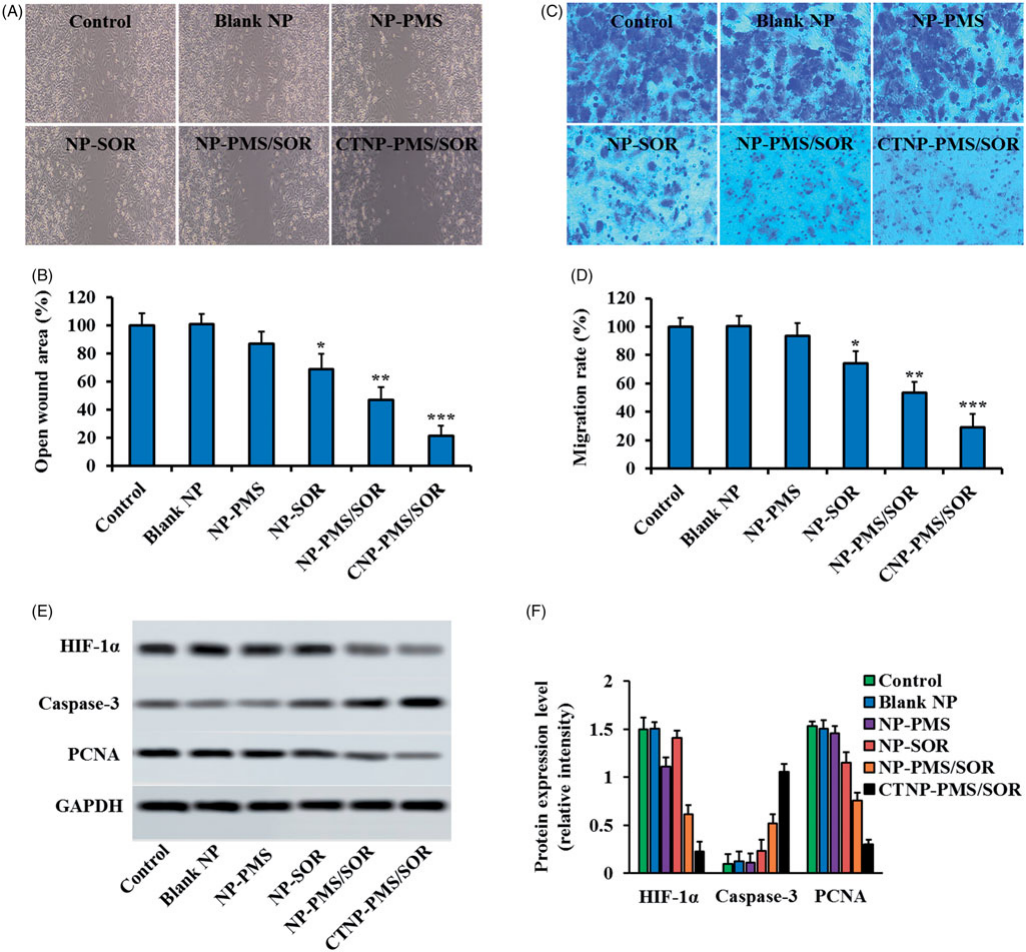
Effects of CTNP-PMS/SOR on HepG2/SOR cell migration and invasion *in vitro.* (A) Wound healing images after scratch respectively incubated with blank NP, NP-PMS, NP-SOR, NP-PMS/SOR, and CTNP-PMS/SOR for 24 h. (B) Quantitative analysis wound healing rate with different treatments. (C) Images of migrated cells stained by crystal violet after treatment with various formulations, obtained by the fluorescent microscope. (D) Quantitative analysis of the invasion rate with different treatments. Qualitative (E) and quantitative (F) analyses of the levels of HIF-1α, Caspase-3, PCNA in HepG2/SOR cells post various treatment. The cells incubated with medium were acted as the control. *p<.05, **p<.01, ***p<.001 significantly lower or higher than the control group.

As demonstrated in Figure 4(A,B), the cells treated with the free SOR exhibited no significant decrease in in the wound healing ability when compared with the control. However, signally slower rate of wound healing was obtained after the SOR was loaded in to the nanoparticles, with a healing rate of 81%. Although the NP-PMS results in no obvious inhibition effect on the cell migration, the migration inhibition ability of NP-SOR was markedly enhanced after the nanoparticle was co-loaded with PMS, with the healing rate was 56%. Additionally, functionalization of CT peptides on the surface of NP-PMS/SOR leads to a dramatical enhancement on the migration inhibition ability of HepG2/SOR cells, with a lowest healing rate of 27%.

For the invasion assay, the obtained results were similar to above results with the cells treated with NP-PMS/SOR exhibiting less amounts of crystal violet compared with the cells incubated with NP-SOR (Figure 4(C)). Additionally, the invasive cells were not significantly decreased post treating by free SOR or NP-PMS while not the NP-SOR. Compared to above groups, the cells treated with CTNP-PMS/SOR displayed the lowest invasion rate, suggesting that the peptides decorated nanoparticles possess the highest anti-metastatic ability. Further quantitative analysis revealed that the NP-PMS/SOR resulted in an invasion rate of 49% while the free SOR, NP-PMS, and NP-SOR was 96%, 94%, and 77%, respectively. After modification of CT peptides, the invasion rate of NP-PMS/SOR was down-regulated to 28% (Figure 4(D)).

### CTNP-PMS/SOR inhibited cell growth, invasion, and migration by inducing cell apoptosis and down-regulation of HIF-1α level

Protein evaluation displayed that the cells treated by PMS have a significant lower expression of HIF-1α than the control group while not the SOR, indicating that the PMS is able of down-regulating the levels of HIF-1α in the SOR-resistant HepG2/SOR cells. Additionally, further investigation revealed that the levels of PCNA, which facilitate to the proliferation of cancer cells, in the cells incubated with SOR while not the PMS is dramatically down-regulated. In contrast, the pro-apoptotic protein, cleaved caspase-3 (C-caspase-3), exhibited an obvious elevation of expression after treated by SOR while not the PMS (Figure 4(E,F)).

### Bio-distribution of the developed nanoparticles

**Figure 5.**
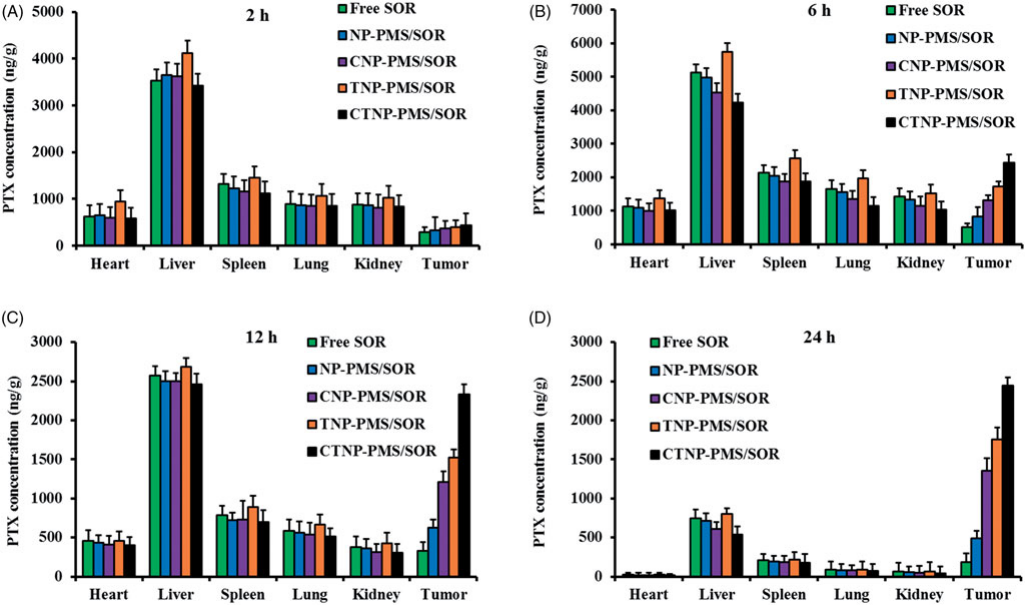
Concentration of SOR in tumor tissue and normal organs of HepG2/SOR cancer-bearing mice at (A) 2 h, (B) 6 h, (C) 12 h, and (D) 24 h, after intravenous administration with free SOR, NP-PMS/SOR, CNP-PMS/SOR, TNP-PMS/SOR, and CTNP-PMS/SOR micelles at a dose of 10 mg/kg SOR. Data were presented as mean ± SD.

The tumor targeting efficacy of peptides functionalized nanoparticles was evaluated in tumor-bearing mice by determining the bio-distribution of drugs. As shown in Figure 5, the distribution of free SOR in normal tissues was dramatically decreased by loading it into the inner core of nanoparticles. Besides, there was no significant difference in drug distribution between the groups of NP-PMS/SOR, CNP-PMS/SOR, and CTNP-PMS/SOR at 2 h after injection. However, the mice injected with TNP-PMS/SOR resulted in highest accumulation of drugs at normal organs. It was mainly owing to the superior cell penetrating capacity and the non-selectivity of TAT peptide. Interestingly, the distribution of drugs at tumor sites was significantly different in various groups after 6 h of injection, with the CTNP-PMS/SOR resulted in the most amount of drugs were detected at tumor tissues and then followed by TNP-PMS/SOR, CNP-PMS/SOR, and CNP-PMS/SOR. After 12 h of injection, the differences in drug distributions of various groups at tumor site were further amplified. Importantly, the total amount of drugs at other orangs is signally down-regulated compared with that of 6 h after injection.

### *In vivo* anti-tumor effect of CTNP-PMS/SOR

**Figure 6.**
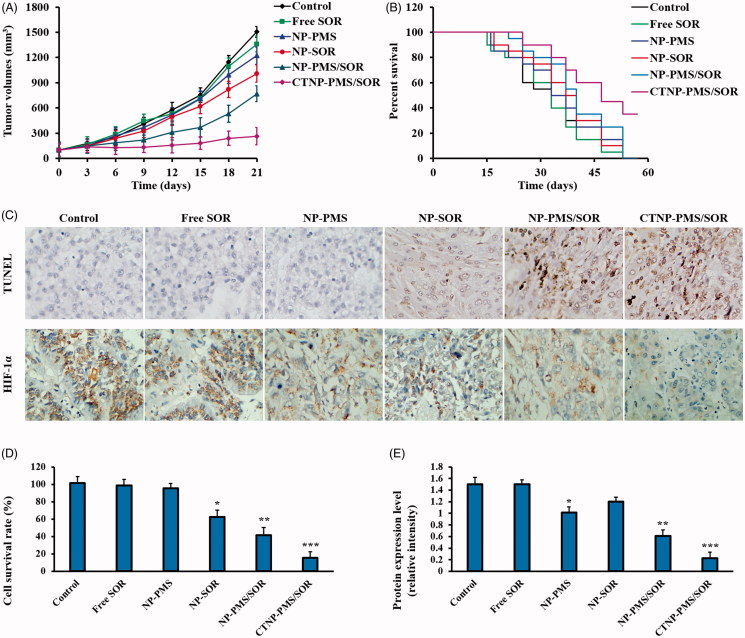
Evaluation of *in vivo* anti-tumor efficacy of different SOR formulations after *iv* injections of free SOR, NP-PMS/SOR, CNP-PMS/SOR, TNP-PMS/SOR, and CTNP-PMS/SOR at a dose of 10 mg/kg SOR. (A) The Kaplan–Meier survival curve of tumor-bearing mice post various treatment. (B) Changes in tumor volumes of mice after treatment. (C) Cell apoptosis and HIF-1α expression in HepG2/SOR tumor tissues determined by TUNEL assay and immunohistochemical staining experiment, respectively. (D) Semi-quantitative analysis of cell apoptosis in tumor tissues induced by different formulations. (E) Semi-quantitative analysis of the HIF-1α expression in HepG2/SOR tumor tissues by western blot assay. *p<.05, **p<.01, ***p<.001 significantly lower or higher than the control group.

Giving the excellent cytotoxicity of CTNP-PMS/SOR to the drug-resistant cancer cells, we subsequently determined the anticancer effectiveness of such nanoparticle on HepG2/SOR cancer-bearing mice. As shown in Figure 6(A), the mice treated by dual-agents loaded nanoparticles (NP-PMS/SOR) exhibited dramatically slower tumor growth than that treated by monotherapy of NP-SOR or NP-PMS. Similar to the results that observed in cellular experiments, the anti-tumor effect was further signally enhanced after functionalization on the surface of NP-PMS/SOR with CT peptides. Survival time of the tumor-bearing mice after treated with various formulations was further evaluated by the Kaplan–Meier survival analysis. As the Kaplan–Meier curves exhibited that the mice injected with CTNP-PMS/SOR achieved the longest median survival time. No significant difference was observed between the control group and the mice treated by NP-SOR or NP-PMS. Similar to above, the survival time of mice was markedly prolonged after treated by NP-PMS/SOR (Figure 6(B)).

### CTNP-PMS/SOR led to high rate of HepG2/SOR cell apoptosis by decreasing the expression of HIF-1α

Cell apoptosis of HepG2/SOR tumor tissues after different treatments was subsequently investigated by H&E staining assay. As shown in Figure 6(C,D), the mice given with NP-SOR or NP-PMS exhibited unconspicuous apoptotic cells in the tumor tissues than the control. However, the area of cell apoptosis in the tumors of mice treated by NP-PMS/SOR was significantly enlarged, confirming the superiority of such combination therapy strategy. Moreover, CTNP-PMS/SOR resulted in more tumor cell apoptosis than the unmodified nanoparticles, indicating the CT peptide is able to mediate more accumulation of nanoparticles at tumor site. Further immunohistochemical staining experiment (Figure 6(C)) and western blot assay (Figure 6(E)) were performed to respectively qualitative and semi-quantitative analyses of the role of PMS in regulation of HIF-1α levels. As confirmed that the cells treated by NP-PMS while not the NP-SOR exhibited down-regulation of HIF-1α expression in HepG2/SOR tumors compared with the control. Moreover, the levels of HIF-1α in the NP-SOR treated tumors could be downregulated by co-treating with PMS. After decoration with the NP-PMS/SOR with CT peptides, the effect of combination effect of PMS and SOR on inducing cell apoptosis and down-regulation of HIF-1α level was further dramatically enhanced.

## Discussion

Sorafenib, the multi-kinase inhibitor, is the first-line systemic therapy approved in HCC (Thomas et al., 2014). As the inhibitor of the Ras/Raf/MAPK and VEGFR/PDGFR signaling pathway, SOR is able to suppress tumor growth and angiogenesis, thereby delaying HCC progression with the prolongation of the patients' survival for almost 3 months (Tanoglu & Karagoz, 2014). However, the clinical application of SOR is largely limited by chemoresistance (Long et al., 2019). It has been uncovered that HIF-1α deficiency impaired SOR resistance induced by PFKFB3 overexpression in HCC cells (Long et al., 2019). Therefore, it indicated that down-regulating the HIF-1α levels may offer a novel therapeutic approach to block SOR resistance of HCC patients.

As a known extract from Herba Plantaginis, PMS has been confirmed to possess anti-cancerous effect on many types of drug-resistant cancers through complex mechanisms (Pei et al., 2015). Based on this, the PMS and SOR were co-entrapped into the hydrophobic core of PLA nanoparticles for combating the chemoresistance of HCC in the present study. The effect of such combination therapy strategy on HCC was thoroughly evaluated and the role of PMS in regulating the expression level of HIF-1α was also investigated here.

In our study, the PMS and SOR dual-loaded nanoparticles (NP-PMS/SOR) was first prepared and decorated on its surface with a multifunctional peptide CT, which is tumor-homing and cell membrane penetrable. Characterization of the developed nanoparticles revealed that it exhibited an almost spherical morphology and core–shell structure. Importantly, decoration of CT peptide on the surface of NP-PMS/SOR did not significantly influence the physicochemical properties of nanoparticles. Besides, drug release behavior of PMS and SOR in two kinds of medium confirmed that the CTNP-PMS/SOR exhibited similar and controlled release pattern.

Affinity of the CT peptides modified nanoparticles to HepG2/SOR cells was evaluated *in vitro* using FITC as the fluorescence probe. Observation of the fluorescence image demonstrated that TNP-FITC treated cells have a more accumulation of nanoparticles within cytoplasm compared with the CNP-FITC treated ones, confirming a superior cell penetrating ability of TAT peptide. However, cells (pH 7.4) incubated with CTNP-FITC displayed a similar fluorescent intensity to the cells treated by CNP-FITC, indicated that the cell penetrating ability of TAT was significantly down-regulated after sealing its functional group by conjugation with C peptides. Interestingly, the cell penetrating ability of TAT was dramatically recovered under the condition of pH 6.0, indicating the linker between the C and TAT peptides is acidic-sensitive enough.

The acidic-dependent HepG2/SOR cancer targeting effects of CTNP-PMS/SOR was further evaluated by *in vivo* bio-distribution of the functionalized nanoparticles. Quantitative results revealed that significant lower accumulation of unmodified nanoparticles was observed in tumor site than the peptides-modified ones. For comparing the groups of TNP-PMS/SOR and CNP-PMS/SOR, it was observed that obviously larger amount of nanoparticles is accumulated at tumor tissues by injection with TNP-PMS/SOR than the CNP-PMS/SOR. However, more accumulation of TNP-PMS/SOR in normal tissues was also achieved, which was mainly due to the unselective property of TAT peptides. Previous studies pointed out that sealing of the fourth lysine of TAT could largely decrease the transmembrane transport capacity of TAT (Liu et al., 2014). In this case, we conjugated the fourth lysine of TAT with a tumor homing peptide CVNHPAFAC via a pH-sensitive hydrazone tendon in the present study. From the results of bio-distribution of various nanoparticles, we could observe that the unselective accumulation of TNP-PMS/SOR in normal tissues was dramatically down-regulated while markedly enhanced the accumulation at tumor sites after conjugated with CVNHPAFAC.

Improved tumor cellular internalization of drugs is supposed to be able of leading to an anticipated enhanced anti-cancerous effect. In accordance with such hypothesis, significantly improved cytotoxicity to HepG2/SOR cells was achieved for CTNP-PMS/SOR (IC_50_ 4.34, 3.05, and 2.16 times lower than that of NP, CNP-PMS/SOR, and TNP-PMS/SOR). The improved anti-cancer efficacy of the CTNP-PMS/SOR was further confirmed *in vivo* on the HepG2/SOR cancer-bearing mice after *iv* injection with various formulations. It was clearly confirmed that the mice treated by CTNP-PMS/SOR achieved the best anti-cancerous effect compared with other groups, with the tumor growth of the mice treated by CTNP-PMS/SOR was largely inhibited. Further TUNEL analysis revealed that the tumor tissues of mice injected with CTNP-PMS/SOR exhibited the largest area of cell apoptosis. These findings together provided robust evidence for the targeting therapeutic effects of CTNP-PMS/SOR.

In generally, the motility of tumor cells was considered as an important measurement for evaluating the metastatic potential of cells. Therefore, in our study, the wound healing assay was used to investigate the lateral migration of cells, while transwell assay focused on the vertical mobility.

As demonstrated in our study, the cells treated with the free SOR exhibited no significant decrease in the wound healing ability when compared with the control. However, signally slower rate of wound healing was obtained after the SOR was loaded in to the nanoparticles, with a healing rate of 81%. Although the NP-PMS results in no obvious inhibition effect on the cell migration, the migration inhibition ability of NP-SOR was markedly enhanced after the nanoparticle was co-loaded with PMS, with the healing rate was 56%. Additionally, functionalization of CT peptides on the surface of NP-PMS/SOR leads to a dramatical enhancement on the migration inhibition ability of HepG2/SOR cells, with a lowest healing rate of 27%.

For the invasion assay, the obtained results were similar to above results with the cells treated with NP-PMS/SOR exhibiting less amounts of crystal violet compared with the cells incubated with NP-SOR. Additionally, the invasive cells were not significantly decreased post treating by free SOR or NP-PMS while not the NP-SOR. Compared to above groups, the cells treated with CTNP-PMS/SOR displayed the lowest invasion rate, suggesting that the peptides decorated nanoparticles possess the highest anti-metastatic ability. Further quantitative analysis revealed that the NP-PMS/SOR resulted in an invasion rate of 49% while the free SOR, NP-PMS, and NP-SOR was 96%, 94%, and 77%, respectively. After modification of CT peptides, the invasion rate of NP-PMS/SOR was down-regulated to 28%.

Finally, the underlying mechanisms were studied. Protein expression studies exhibited that there was a higher expression of HIF-1α in SOR-resistant HepG2/SOR cells and tumor tissues. Our results showed that the expression level of HIF-1α increased in hypoxia microenvironment, which could be significantly decreased after PMS while not the SOR. Similar results were observed in subcutaneous xenotransplanted tumor. The expression level of HIF-1α was signally higher in the untreated tumor tissues, whereas its expression was markedly decreased after PMS treating. These results together, revealed that the PMS treatment could reverse the expression changes of HIF-1α induced by hypoxia.

## Conclusions

In the present study, we have developed a multi-functional nanoparticle, which is able of co-loading with SOR and PMS for combating the drug-resistance of HCC. To enhance tumor accumulation of drugs, the polypeptide CT, which is tumor-homing and cell membrane penetrable, was further decorated on the dual-agents loaded nanoparticles (CTNP-PMS/SOR). Results of cellular experiments and *in vivo* assays revealed that co-loaded with PMS significantly enhanced the anti-cancerous effect of SOR on malignant HCC. Further mechanism studies confirmed that the PMS enhanced the anti-tumor effect of SOR on the drug-resistant HCC through reprograming the tumor hypoxic microenvironment. Moreover, decoration of CT peptides on the surfaces of NP-PMS/SOR resulted in more accumulation of drugs at tumor sites and penetration of agents into tumor cells, which in turn led to stronger capacity of tumor growth inhibition.
